# SnIPRE: Selection Inference Using a Poisson Random Effects Model

**DOI:** 10.1371/journal.pcbi.1002806

**Published:** 2012-12-06

**Authors:** Kirsten E. Eilertson, James G. Booth, Carlos D. Bustamante

**Affiliations:** 1 Bioinformatics Core, J David Gladstone Institutes, San Francisco, California, United States of America; 2 Department of Biological Statistics and Computational Biology, Cornell University, Ithaca, New York, United States of America; 3 Department of Genetics, Stanford University, Stanford, California, United States of America; University of California San Diego, United States of America

## Abstract

We present an approach for identifying genes under natural selection using polymorphism and divergence data from synonymous and non-synonymous sites within genes. A generalized linear mixed model is used to model the genome-wide variability among categories of mutations and estimate its functional consequence. We demonstrate how the model's estimated fixed and random effects can be used to identify genes under selection. The parameter estimates from our generalized linear model can be transformed to yield population genetic parameter estimates for quantities including the average selection coefficient for new mutations at a locus, the synonymous and non-synynomous mutation rates, and species divergence times. Furthermore, our approach incorporates stochastic variation due to the evolutionary process and can be fit using standard statistical software. The model is fit in both the empirical Bayes and Bayesian settings using the lme4 package in R, and Markov chain Monte Carlo methods in WinBUGS. Using simulated data we compare our method to existing approaches for detecting genes under selection: the McDonald-Kreitman test, and two versions of the Poisson random field based method MKprf. Overall, we find our method universally outperforms existing methods for detecting genes subject to selection using polymorphism and divergence data.

## Author Summary

We present a new methodology, SnIPRE, for identifying genes under natural selection. SnIPRE is a “McDonald-Kreitman” type of analysis, in that it is based on MK table data and has an advantage over other types of statistics because it is robust to demography. Similar to the MKprf method, SnIPRE makes use of genome-wide information to increase power, but is non-parametric in the sense that it makes no assumptions (and does not require estimation) of parameters such as mutation rate and species divergence time in order to identify genes under selection. In simulations SnIPRE outperforms both the MK statistic and the two versions of MKprf considered. We then apply our method to *Drosophila* and human-chimp data.

## Introduction

### Background

Populations evolve over time and how they evolve is the product of different evolutionary forces. Population genetic theory gives us mathematical descriptions of how each of these forces is thought to affect the patterns of genetic variability within and between species. However, if the goal is not to start with an evolutionary model and see what happens, but rather to start with the data and understand what caused it one usually encounters an identifiability issue. For this reason, most population genetic data analyses looking for mutations under selection start by assuming a neutral population genetics model (constant population size, panmictic population, no migration), and test for deviations from this model. Commonly used examples of such procedures include tests based on summary statistics of the site frequency spectrum (distribution of mutation frequencies), such as Tajima's D [1]. However, since demographic factors (eg population growth) also effect the site frequency spectrum these tests are usually inconclusive. Tests based on linkage disequalibrium are also quite sensitive to demography as well as assumptions on recombination rates [2]. The HKA statistic [3] makes use of divergence data as well as within species variation by estimating the variance of divergence to polymorphism ratios among loci. However, migration will result in a high variance of coalescent times among the loci, making the HKA test also sensitive to demography [4]. See Nielsen 2005 [2], for an excellent review of these procedures.

One class of tests which is generally robust to demography are those tests commonly referred to as “McDonald-Kreitman-type tests” [4]. This class includes the McDonald-Kreitman (MK) test [5] as well as MKprf [6]. The theory behind the MK test is developed in the following section.

Unlike many of the tests mentioned above, the method we present here assumes no particular population genetic model - in other words it is a non-parametric approach. Similar to the MK statistic, it is also generally robust to demography. Our method, which we call SnIPRE for *S*electio*n I*nference using *P*oisson *R*andom *E*ffects, works by modeling the variation within and between species as a combination of four types of “effects”, one for each class of variation. These effects are functions of unknown population parameters of interest, including the selection coefficients.

Previously, we have developed a suite of powerful approaches that can estimate the average strength of selection operating on a locus and/or the distribution of fitness effects under a specified population genetic setting for MK polymorhism and divergence data (see [7]–[12]). A main advantage of the “MKprf” approach is that it is much more powerful than carrying out individual MK tests and then correcting for multiple tests. A perceived disadvantage to some investigators is that it requires specifying a population genetic model and then fitting the parameters of that model. Some investigators have also been concerned about the use of Bayesian priors on the distribution of effects and the impact these can have on inference [13].

There are two main advantages of SnIPRE over MK and MKprf, which we highlight here. The first is that it can reliably identify genes under weak and strong negative as well as positive selection without needing to specify a population genetic model a priori. Nonetheless, because it “borrows information” from the rest of the genome regarding the average and variance in polymorphism to divergence, it outperforms the one-at-time MK test. This gain in power is attributable to SnIPRE's use of a “James-Stein” class of estimator. The second advantage is that if one is willing to assume a particular population genetic model, it is possible to view the SnIPRE parameters as a re-parameterization of the population genetic model. With these additional assumptions, we can extend our inference beyond idenfication of genes that are not evolving according to the neutral theory, to quantification of strength and directionality of the selection forces.

In this paper we will develop the model and the interpretation of its terms, and then describe how that model can be fit in both the empirical Bayes (SnIPRE) and fully Bayesian (B SnIPRE) settings. We also show how this model is robust to demographic history and recombination using standard coalescent simulations. Furthermore, we demonstrate how the Poisson Random Field estimates of average selection intensity, species-split time, mutation rate, and degree of selective constraint at the locus can be “extracted” directly from the SnIPRE estimates. We then compare the SnIPRE methods to the MK statistic and MKprf methods in detecting and estimating selection and other population parameters in simulations, and apply SnIPRE to data from a *Drosophila* comparison and human-chimp comparison.

### The MK statistic

Because SnIPRE works by picking up on the same type of signature of selection as the MK statistic, we will start with a review of this method and the theory behind it. While most techniques to identify loci under selection require assumptions about demography (particularly constant population size and no substructure), the MK statistic does not. Like the HKA statistic, it works by comparing divergence information between inferred neutral sites (such as synonymous sites in a protein-coding gene) and sites potentially under selection (such as non-synonymous sites at the same gene). Strictly speaking, the test is a test of the neutral protein evolution hypothesis which states that the vast majority of evolutionary changes at the molecular level are caused by random drift of selectively neutral mutants (not affecting fitness) [14]. Although very tempting, the test itself does not allow for inference about the type of selection (negative, positive, or balancing). For example, as noted in original paper, negative selection in recently expanding populations may appear as positive selection. Thus, without additional assumptions on population dynamics the direction cannot be inferred. There have been notable extensions to the MK test, including using non-coding sites whereby upstream regions of a gene are compared to neighboring introns or synonymous sites [15]. Another extension is the estimator 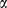, [16] which estimates the the proportion of amino-acid substititutes which are driven by adaptive selection. These extensions, and the additional set of assumptions they require, are not considered here.

In its traditional form the MK table consists of counts for four categories of mutations which occur in the coding region of a gene: polymorphic synonymous, divergent synonymous, polymorphic non-synonymous, and divergent non-synonymous, see Table 1. A mutation that occurs in every individual in the sample from one species is considered divergent, otherwise considered polymorphic. A mutation that occurs where it changes the amino acid produced is considered non-synonymous, otherwise considered synonymous. If the mutations are neutral, one would expect the ratio of polymorphic synonymous (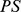) to divergent synonymous (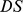) mutations to be the same as the ratio of polymorphic non-synonymous (*PN*) to divergent non-synonymous (*DN*) mutations, 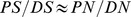. If this is not true, then we are seeing either an excess of 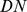 mutations, or shortage 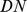 mutations. Intuitively, it makes sense to consider an excess of 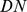 as evidence supporting positive selection as it appears that mutations that change the amino acid are being fixed in the population at a higher rate. Alternatively, a shortage of 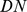 could be considered as evidence of negative selection as it would appear as though mutations that change the amino acid are being fixed at a lower rate. This interpretation of the data is fairly straightforward considering an additive model of selection with stationary population sizes. However, as mentioned above and as discussed in [17], asessment of directionality from the MK statistics should be used with caution as it is sensitive to changing population dynamics. It should be noted, however, that in the case of strong negative (i.e. purifying) selection, the signature will be less clear in an MK table since mutations are not likely to segegrate in the population long enough to contribute to the polymorphism count. Thus, in the case of strong negative selection a reduction in the number of both polymorphic and divergent non-synonymous mutations is to be expected, and the MK test will have reduced power to detect this type of selection.

**Table 1:** MK table.

	MK		SnIPRE		
	Polymorphic	Divergent	Polymorphic	Divergent	
Synonymous	PS	DS	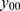	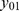	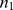
Non-Synonymous	PN	DN	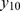	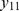	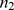
		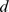			

Notation used for the MK statistic and SnIPRE. 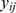 = the number of mutations a gene has in category 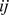; 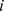 = 1 if the mutations are non-synonymous, 0 otherwise; 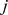 = 1 if the mutations are divergent, 0 otherwise.

McDonald and Kreitman [18] use Fisher's exact test of independence on MK tables to identify genes under selection. This test can be justified using coalescent theory where we have the additional assumptions of i) no recombination within a gene ii) all mutations are selectively neutral [19]. In this setting, the MK test constitutes a test of this second assumption. Under the coalescent theory model, mutations are Poisson distributed across a gene genealogy with expected value 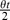 across a geneology of length 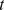, where 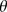 is the mutation rate. Thus, conditioning on the total mutations (sufficient statistic for tree length) we have that
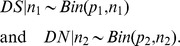
We wish to test 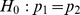, the probability that a synonymous mutation appears fixed is the same as the probability that a non-synonymous mutation appears fixed in the sample. Under this null hypothesis, 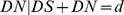 follows a hypergeometric distribution with parameters, (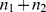, 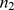, 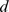).
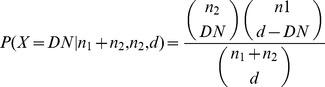
As long as the non-synonymous and synonymous sites are interspersed among each other, they will be similarly affected by demography and have the same distribution of coalescent times, thus the test is robust to demography.

Motivated by the MK statistic, the SnIPRE framework uses the MK table polymorphism and divergence data for identifying genes under selection. Using generalized linear mixed models we incorporate genome wide effects into our analysis as fixed effects, and individual gene effects as random effects. This method allows us to pool information across genes which increases our power to detect those under selection.

MKprf is another method that was developed by us which directly estimates the posterior distributions of genomic parameters, such as the species divergence time, based on the MK tables' synonymous cell entries. The posterior of the selection coefficients for each gene are then calculated conditional on these genomic parameters and the non-synonymous cell entries in the MK table, see [7].

## Methods

### Data

The data consists of MK table counts for each gene, as well as the total number of synonymous sites and non-synonymous sites surveyed. Incorporating the number of sites into our model allows us to extend our inference beyond non-synonymous and divergent interaction effects to include effects due to changes in the mutation rate, both in the synonymous and non-synonymous sites.

### Model

Let 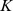 be number of genes in the sample. Thus we have 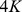 mutation counts 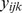, where 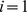 if the mutation is non-synonymous, 0 otherwise, 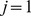 if the mutation is fixed in the sample among the two populations being compared, 0 otherwise, and 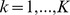 according to gene identification number. The mutation counts are assumed to be Poisson distributed, 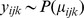, conditional on the covariates. The log of the expected mutation count is modeled using a generalized linear mixed effects model. The fixed effects include an intercept, an effect if the mutation is non-synonymous, an effect if the mutation is fixed, and an interaction effect if the mutation is both fixed and non-synonymous. Additionally the model includes four random effects: a gene effect, and the two-way and three-way interactions between the gene, non-synonymous, and divergence effects. An offset term is used to control for the number of sites sampled in the gene where a mutation of type 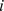 could occur, 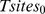 for synonymous mutations, 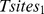 for non-synonymous mutations.
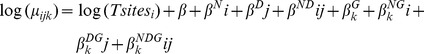
(1)


By using fixed and random effects in the model we are assuming that these gene-specific effects come from some distribution, and that distribution is estimated from the data. The use of mixed effects is particularly relevant in this setting where it capitalizes on the fact that genes share a phylogeny. Thus, even though the mutation rate, coalescent times, constraint and selection forces will vary across genes, the distribution of the influence of these forces across genes can be well estimated by viewing the data set as a whole. From this perspective we estimate the fixed effect terms (genome-wide average estimates) of our model, as well as the variability in the distribution in of the random (gene-specific) effect terms of the model. The random effects, or gene-specific parameters, are then estimated given this context. Below we describe how the terms in this model allow us to estimate for any given gene the average effect of mutation, divergence, constraint and selection levels over time.

Of primary interest is identifying genes under selection, either positive or negative. Identification of these genes can be done quite easily in the SnIPRE framework with only the assumptions of the MK test: i. synonymous and non-synonymous sites sampled are interspersed; ii. synonymous sites are not under selection. The non-synonymous-divergent interaction effects, 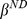 and 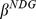, capture an average genome-wide selection effect and the gene-specific selection effects. The gene-specific selection effect 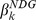 for a particular gene 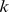, captures how the 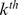 gene varies from the average selection effect, 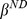, of all genes included in the sample. The 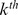 gene's selection effect relative to neutrality is reflected in the sum of these two interaction terms, 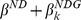. Thus, we refer to 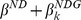 as the *selection effect* for the 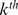 gene. For example, an estimated 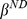 of greater than 0, say 0.5, means that the expected selection coefficient for a gene from that data set is positive. A gene-specific selection effect, 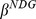, may be negative, say −0.3, indicating that the estimated selection effect for that gene is lower than the average for genes in the data set. The estimated selection effect on that gene relative to neutrality (zero being neutral) is the sum of these two effects. In this example, the estimate would be positive, 0.5+(−0.3) = 0.2.

The other terms in the SnIPRE model are also quite interpretable. The interecept and the gene specific effect, 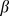 and 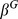 reflect the mutation rate. Here again the 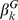 term captures how the mutation rate for the 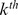 gene varies from the average mutation rate of the genes in the sample, 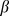. We refer to 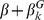 as the *gene effect*. Similarly, 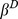 and 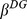 reflect divergence time, and 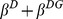 is referred to as the *divergence effect*. The proportion of non-synonmyous mutations that are non-lethal are reflected in 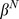 and 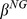. We refer to 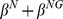 as the *constraint effect*. These relationships are summarized in Table 2. A precise relationship between these model parameters and the evolutionary parameters that influence them is defined the Poisson Random Field framework and discussed in the next section. Examples of the interpretation of these model parameters is provided in the application section.

**Table 2:** SnIPRE coefficients and population genetic parameters.

Terms	Related parameters
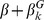	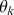 , mutation rate for the 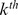 gene
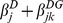	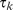 , divergence time for the 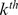 gene
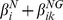	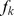 , proportion of non-synonymousmutations that are non-lethal for the 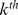 gene
	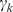 , selection coefficient for 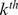 gene
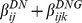	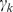 , selection coefficient for 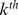 gene
	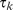 , divergence time for 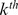 gene

Summary of the relationship between SnIPRE coefficients and population genetic parameters.

**Figure 1:**
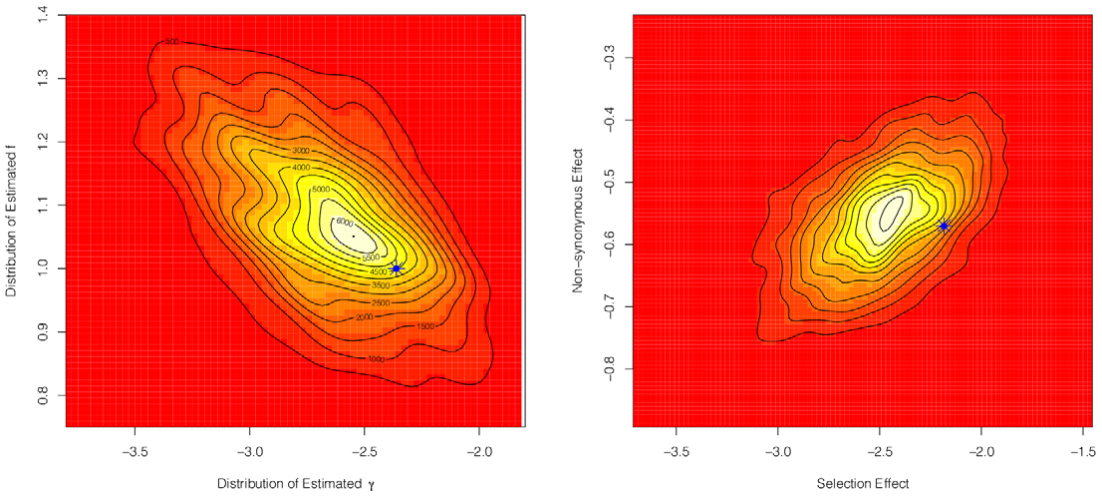
Example joint distribution of the estimated selection effect and the constraint effect for a particular gene. Data simulated using PRFREQ. The blue asterisk denotes the true location of parameters.

We fit this model in R [20] using the lme4 package [21], and a Bayesian implementation is also fit using WinBUGS [22], [23]. In the Bayesian setting (B SnIPRE) we construct credible intervals for these effects based on the MCMC samples (other packages may be used instead to fit the model, e.g. the R package MCMCglmm [24] or JAGS [25]). In the empirical Bayes setting (SnIPRE) confidence intervals are constructed for the random effect estimates based on the standard errors. When fitting SnIPRE using the lme4 package we specified a general (unstructured) covariance. Using a structure other than a general covariance structure presupposes a functional form, e.g. a covariance matrix with the off-diagonal elements all zero would indicate that the gene specific effects are independent of each other. Incorrectly assuming a particular form would lead to spurious results, and the the property of best linear unbiased estimates would no longer hold for the model coefficients. While inference would be more powerful if the correct form of the covariance matrix was known, the unstructured covariance allows for conservative estimation directly from the data. In practice we have found that allowing the general covariance structure versus assuming the random effects are independent of each other greatly improves the fit of the model and improves the prediction of genes under selection. Modeling a general covariance structure makes sense intuitively. For example, for a particular gene the non-synonymous and selection effects are especially likely to be correlated as selection affects the amount of time a non-synonymous mutation exists as a polymorphism before becoming fixed or eliminated. The selection effect reflects the selection coefficient 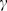, and the non-synonymous effect reflects mutation constraint, 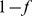. Because of this relationship, one may be interested in examining the joint distribution for these estimated effects for a particular gene. This is easily accomplished in the Bayesian setting using the MCMC chains. As an example, see Figure 1.

For the Bayesian model the fixed effects have Normal priors with mean 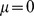, and precision 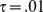. The priors for the random effects for each gene were multivariate normal with mean 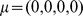, and precision 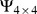. The precision matrix is modeled as a hyperparameter in order to estimate the covariance structrue among the random effects. Using the conjugate prior, the Wishart disribution, we set 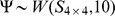, where 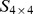 is the identity matrix. Because the mutation counts are low these priors are considered non-informative.

An alternative formulation of the Bayesian model using hierarchical centering maybe be preferable as it results in quicker convergence [26]. In the hierarchical centering formulation the fixed effects appear as hyper parameters about which the random effects are centered. The models are equivalent and as long as convergence criteria are met will yield the same inference.

### Coalescent and Poisson random field frameworks

In standard coalescent theory we have 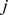 lineages coalescing at time points exponentially distributed with rate equal to 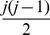. The number of segregating sites follows a Poisson process with rate 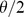 per unit of time. Conditioning on the length of genealogy, 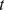, which is a function of the coalescent times, the number of segregating sites is Poisson distributed with mean 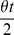. Thus, we have the expected mutation count, 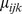, is a function of the sample coalescent times, as well as the mutation rate 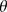 [19]. Additionally, the expected mutation count should be adjusted for constraint, 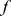, and selection 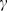. This is consistent with our model where the effects of mutation rate and divergence is estimated from the synonymous mutations, and constraint and selection are estimated from the non-synonymous.

Our model also works well in the Poisson random field (PRF) framework which assumes i. mutations arise at exponentially distributed times, ii. each mutation occurs at a new site, and iii. each mutant follows and independent Wright-Fisher process (no linkage)[27]. SnIPRE can be viewed as a re-parameterization of the PRF framework. Thus it is convenient to use the relationships between the SnIPRE coefficients and the PRF model to obtain estimates of 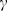 (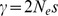, where 1+s is the fitness of mutants, and 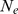 is the effective population size), as well as 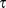, 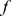, and 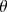 (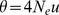 where 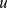 is the nucleotide mutation rate). We can derive the relationship between the population genetic parameters and the SnIPRE coefficients by comparing the predicted MK table counts provided by SnIPRE, see Table 3, which are written in terms of model coefficients, to the theoretical expected MK table counts given in Table 4. These relationships are derived below; 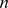 and 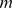 represent the number of samples from the population of interest and the outgroup.

**Table 3:** SnIPRE predicted mutation counts.

	Polymorphic	Divergent
Syn	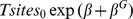	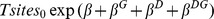
Non-syn	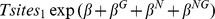	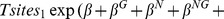
		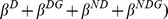

The predicted mutation counts expressed in terms of the number of synonymous and non-synonymous sites sampled 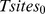, 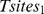, the gene effect 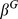, nonsynonymous effect 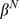, divergent effect 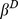, and their interactions.

**Table 4:** Expected mutation counts.

	Polymorphic	Divergent
Syn	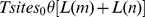	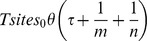
Non-syn	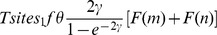	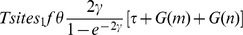

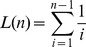

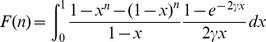

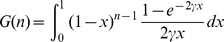

The expected mutation counts expressed in terms of the number of synonymous and non-synonymous sites sampled 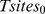, 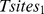, selection coefficient 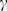, the species-split time 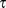, the mutation rate 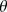, the proportion of lethal non-lethal mutations 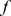, and the number of samples in the population of interest and the outgroup 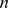 and 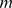, according to the Poisson Random Field framework.

The gene effect 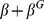, is a function of the mutation rate 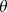.
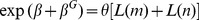
(2)


The divergence effect, 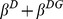, is a function of the divergence time 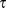.
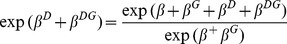
(3)

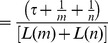
(4)


The selection effect 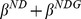, is a function of the selection coefficient 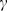, and the time to the most recent common ancestor 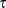.
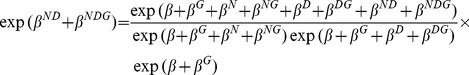
(5)

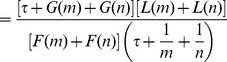
(6)The selection effect reflects the interaction of the non-synonymous and divergent effects on the expected mutation count. Under the PRF framework we assume a neutral demography. Thus, a positive (negative) selection effect corresponds to a positive (negative) selection coefficient. That positive (negative) selection leads to the higher (lower) rate of fixation for non-synonymous mutations makes sense intuitively. A positive selection effect indicates that mutations that are non-synonymous are being fixed at a higher rate than expected under the null hypothesis of no selection. A negative selection effect indicates that mutations that are non-synonymous are being fixed at a slower rate than expected.

The non-synonymous effect, 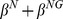, may also be thought of as a constraint effect since it is a function of the proportion of non-synonymous mutations that are non-lethal 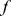, as well as the selection coefficient, 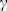.
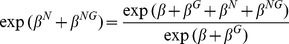
(7)

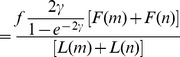
(8)The constraint effect, 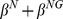, reflects the effect that mutations being non-synonymous (versus synonymous) has on the expected count. A negative (positive) constraint effect indicates that non-synonymous polymorphic mutations are either being fixed or eliminated at a higher (lower) rate than synonymous mutations. Thus, after estimating the selection coefficient to account for the rate at which non-synonymous mutations are fixed, we can estimate from the constraint effect the proportion of mutations that are lethal, and therefore quickly eliminated from the population. While the selection effect is useful for identifying selection on mildly deleterious mutations as well as advantageous mutations, the constraint effect can be used to identify cases of strong negative or purifying selection.

It is interesting to note that these are the relationships used by Sawyer and Hartl (1992) to fit their single locus PRF models to 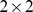 MK data. What is different about our approach is that we do not require a PRF parameterization for inference; rather, it naturally falls out from consideration of the standard log-linear model analysis of multi-way contigency tables. Several of the simulations in the next section are done in the PRF framework using PRFREQ [12]. Also included are several simulations using SFS_CODE [28] that show our estimation of population genetic parameters to be fairly robust to the PRF assumption of no linkage between sites. Specifically, the false positive rate remains low for identification of genes under selection. The primary consequence of linkage is underestimation of the magnitude of selection. We plan to explore these results more in a later paper.

## Results/Discussion

To assess and compare the performance of the SnIPRE methods against the MK statistic and MKprf method we simulated data using 3 different methods. The first method, based on coalescent theory, was implemented in R. The second method, PRFREQ, simulates data based on the PRF framework. The third method is a forward simulation method, SFS_CODE. In these simulations our first goal was to compare the false positive rates of the methods using simulations under neutrality. Additionally, we simulated data with selective constraint but without selection which illustrates SnIPRE's ability to distinguish between mutational constraint and selection. Using PRFREQ, we were also able to simulate data sets with a distribution of selection coefficients and use this to compare the methods in a litany of non-neutral settings.

For the results reported below, the MK test (Fisher's exact test) was applied and the resulting p-value left unadjusted for multiple testing, significance was determined by an 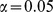 cutoff. For B SnIPRE, and the two versions of the MKprf significance was evaluated based on the posterior distributions; if at least 97.5% of the posterior distribution lay to one side of zero, the estimate was deemed significant. This cutoff was chosen to correspond to a two-sided test at 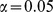 level. For SnIPRE, significance was established based on the standard error and estimate of the effect of interest. A more precise calculation of the significance of an effect is possible in the empirical Bayes framework by estimating the profile likelihood via Laplace approximations. This estimation procedure is not discussed here.

### Simulations under neutrality

To assess false positive rate FPR for each of the methods, we simulated data using standard coalescent theory. In Table 5, we report the false positive rate for a data set with 1,000 neutrally evolving genes simulated from a pair of populations of constant size that split 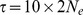 generations ago, with mutation rate 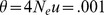. The standard MK approach had an FPR = 0.02. SnIPRE performed very well with an FPR
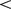
0.001 for both the Bayesian and empirical Bayes approaches. MKprf had mixed performance, depending on assumptions regarding the variance of the distribution of fitness effects. For fixed variance, 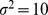, the FPR = 0.14 which is relatively high. This is a mode of MKprf that has a very wide prior distribution that is not updated by information from other loci. When that information is incorporated we see that MKprf (estimated 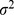) also has a low FPR, 0.012.

**Table 5:** False positive rate.

Method	False Positive Rate
SnIPRE	0.00
B SnIPRE	0.00
MKprf ( 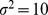 )	0.14
MKprf (estimated 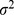 )	0.01
MK	0.02

False positive rate in a data set of 1000 genes simulated using the coalescent method.

Next we investigated the impact of demographic history as well as recombination on the FPR of the methods using the forward simulator SFS_CODE. In Table 6, we report simulation results for 5 demographic settings for 1,000 gene data sets including three bottleneck scenarios, one population growth model, and constant population size. From these simulations we see that both the MK method and SnIPRE methods have very low false positive rates, with the SnIPRE methods performing slightly better. MKprf with estimated variance has similarly very low false positive rates, however MKprf with 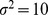 has consistently higher false positive rates. As stated above, all these methods should be robust to demography. This appears to be the case in our simulations as the false positive rates remain consistent for each method across demographies.

**Table 6:** False positive rate and demography.

	Bottleneck 1	Bottleneck 2	Bottleneck 3	Expansion	Constant
SnIPRE	0.00	0.00	0.00	0.00	0.00
B SnIPRE	0.00	0.00	0.00	0.00	0.00
MKprf ( 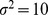 )	0.11	0.08	0.01	0.11	0.13
MKprf (estimated 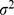 )	0.00	0.00	0.00	0.00	0.03
MK	0.02	0.02	0.01	0.02	0.03

False positive rates when no selection, and under various population growth models.

The key point from all these simulations is that SnIPRE performs just as conservatively as the MK test and better than MKprf under a litany of neutral scenarios that might be cause for concern in analyses for inference of selection.

### Simulations with constraint

A particularly interesting application of SnIPRE is to identify regions of the human (or a new genome) that show very low levels of variation based on both polymorphism and divergence data. These might be interpretable as regions of high selective constraint either at the amino acid or non-coding level (for comparison with a flanking “neutral” standard) and may represent biologically meaningful sequences, see [29], [30].

**Figure 2:**
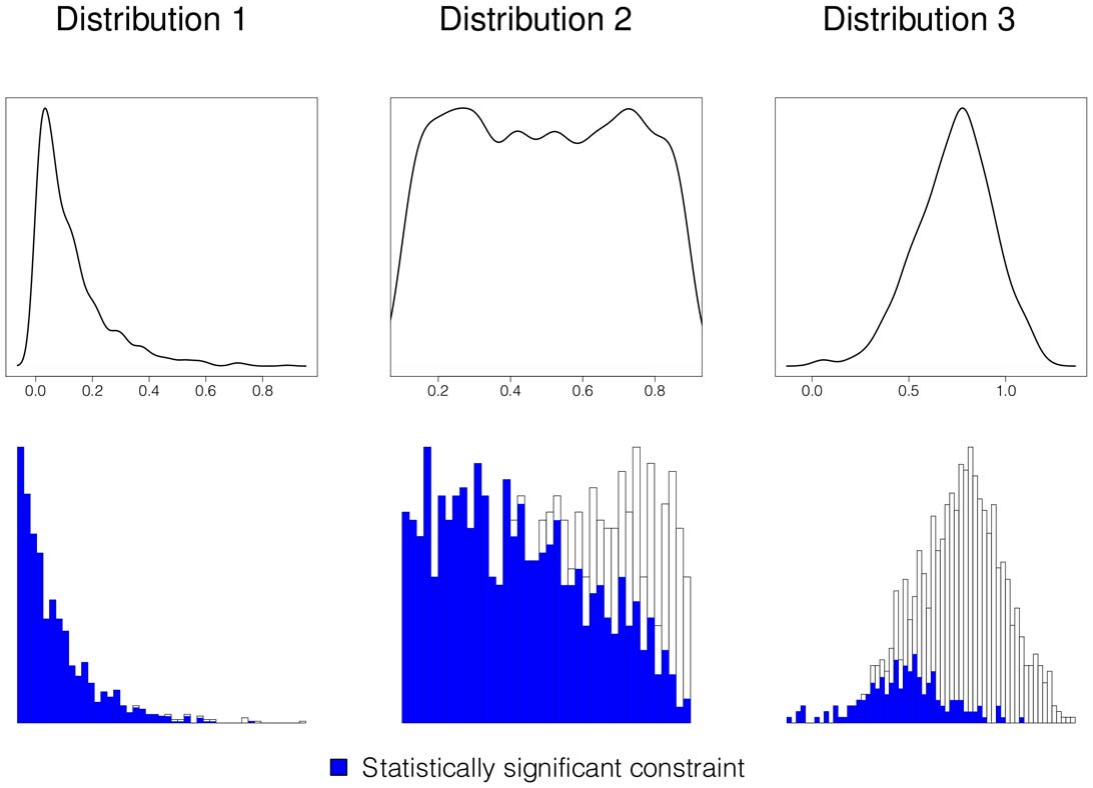
Classification of constraint. Top: Distribution 1, 2, and 3 of 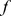 used in the coalescent simulations for Table 7. Bottom: Proportion of constraint effects classified as significant by SnIPRE; x-axis is true proportion of non-lethal mutations, 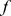.

To quantify the power of SnIPRE to identify constrained loci, we used the coalescent method to simulate three different scenarios with varying degree of selective constraint, or 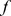, among genes in 1,000 gene data sets. Here we consider the case where some proportion of sites are very strongly constrained (any mutation at these locations is considered lethal), and not the case where the mutations are of weak negative effect and could rise in frequency and contribute to polymorphism (considered in the simulations below). That is, these regions do not exhibit a deviation in polymorphism verus divergence; however, they will be outliers with regard to the genome-wide pattern of overall genetic variation. In Table 7 and Figure 2 we see the results from three coalescent simulations with three different distributions on mutational constraint, 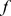. A comparable estimate of constraint from the MKprf methods is a function of its estimated nonsynonymous and synonymous mutation rates 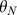, and 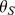:
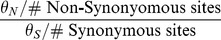
The SnIPRE methods performed quite well on data from distribution one with 98% and 99% correct, the MKprf methods yielded only 43% and 67% correct. Distribution 2 has a wider variety of constraint and presents more of a challenge for both SnIPRE (66% and 86%)and MKprf (38% and 51%) methods. Distribution three contained only mild to moderate constraint and was the most challenging of the three distributions. Here, the B SnIPRE method proved to be the most powerful of the four methods, with 45% correctly classified, and the MKprf methods yeilded approximately 21% correct, and SnIPRE approximately 17% correct. For all three distributions the SnIPRE methods correctly classified the selection effects as neutral. From these results we see that the SnIPRE model is able to detect strong constraint, and can distinguish these effects from those of selection.

**Table 7:** Realized coverage of 95% CI for 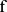 and γ when 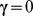, 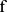 varies, and there is linkage among sites.

	% Correct 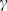			% Correct 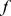		
	Dist 1	Dist 2	Dist 3	Dist 1	Dist 2	Dist 3
SnIPRE	100.0	100.0	100.0	98.7	66.1	17.9
B SnIPRE	100.0	100.0	100.0	99.2	86.0	43.4
MKprf ( 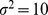 )	69.3	92.2	87.9	43.0	38.7	21.5
MKprf (estimated 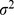 )	71.1	99.3	99.3	67.6	51.6	20.7

Results for coalescent model simulations with a distribution on 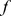, and no selection 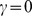. 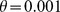.

**Figure 3:**
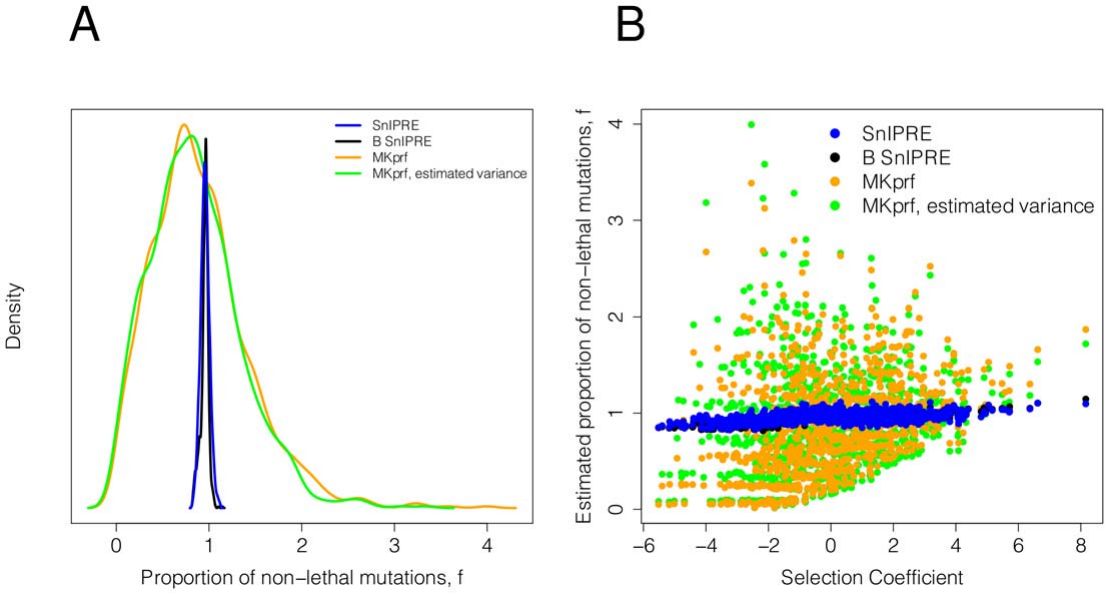
Comparison of estimates of constraint when 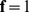 (no constraint). A: The distribution of constraint estimates. B: Constraint estimates versus the selection strength.

A comparison can also be made when selection is present, and there is no constraint (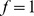). To do this we considered a data set with selection coefficients drawn from a normal distribution with a mean of zero, a standard deviation of two, and with no constraint. In Figure 3 A we see that SnIPRE's estimated constraint effects are quite accurate (very close to one), while the MKprf methods have much more variable estimates. The SnIPRE method's estimates of constraint are somewhat correlated with the selection coefficient, however we see in Figure 3 B that the effect of this trend is minimal.

### Simulations with selection

#### Classification of selection effect

**Figure 4:**
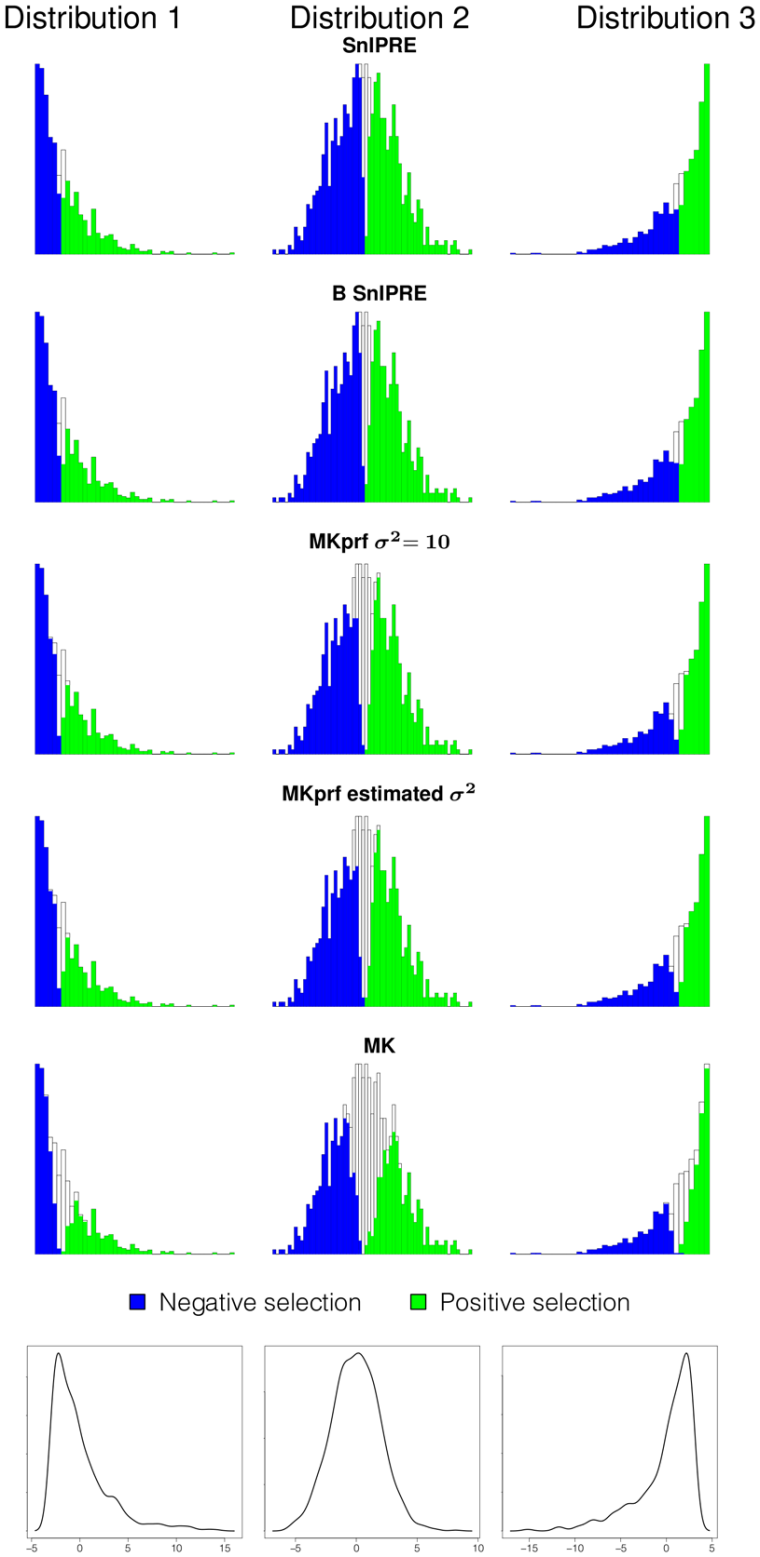
Classification of selection effect for *Drosophila*-like simulations. Shaded regions of histogram represent the proportion of genes under selection classified as under selection; x-axis is true selection coefficient; 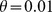.

**Figure 5:**
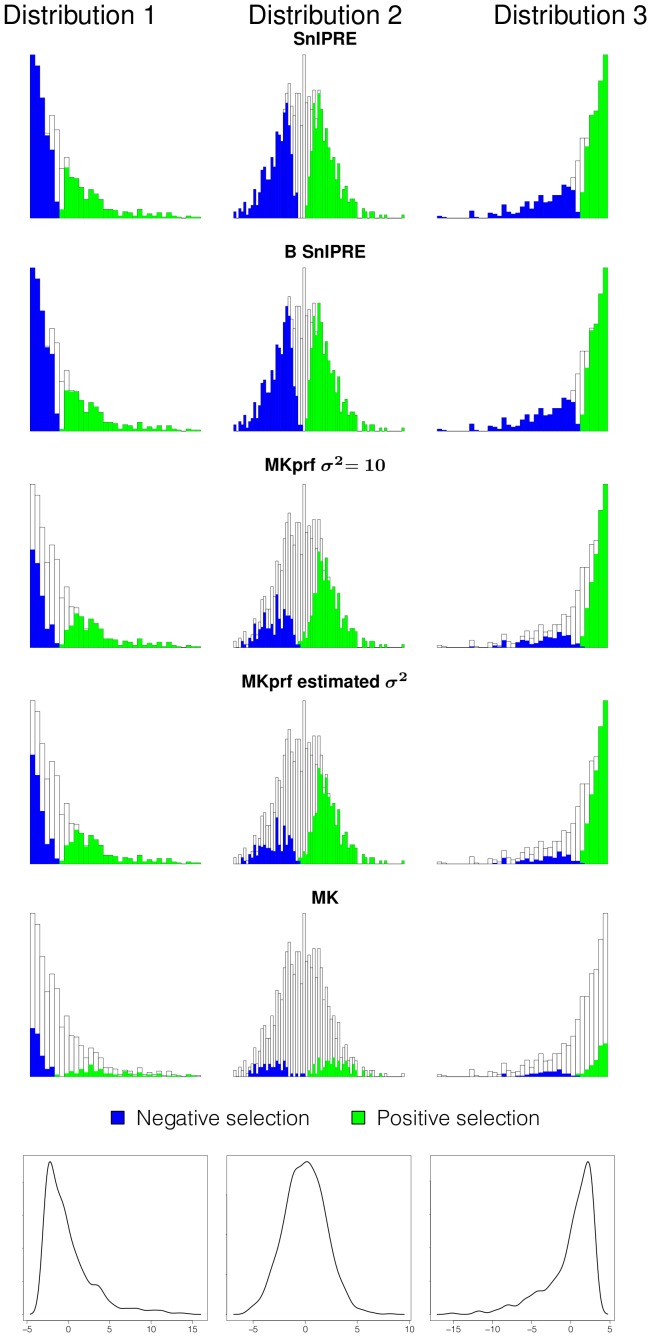
Classification of selection effect for human-like simulations. Shaded regions of histogram represent the proportion of genes under selection classified as under selection; x-axis is true selection coefficient; 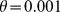.

To assess performance when the selection coefficients come from some distribution, we simulated data using PRFREQ for six data sets of 1,000 genes. Selection coefficients for our simulations are drawn from three distributions, which are shown at the bottom of both Figure 4 and Figure 5. These selection coefficients were then used to simulate data with drosophila-like parameters 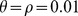, and with human-like parameters 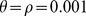. In Figures 4 and 5 each row of histograms illustrates a particular method's performance on data from each of the simulations. The colored portions of the histograms represent the proportion of selection coefficients in each bin correctly classified as under selection, with the true selection coefficient values given along the x-axis. These results are also summarized in Table 8. From our simulations, we found the SnIPRE method to be a dramatic improvement over other methods in identifying genes under selection, especially when table counts are low, as with a human-like mutation rate of 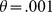. For example, the SnIPRE methods classify 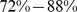 of genes correctly, MKprf methods classify 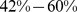 correctly, and the MK statistic just 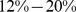 correctly. For the drosophila-like simulations the SnIPRE methods classify 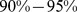 correctly, MKprf methods classify 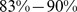 correctly, and the MK statistics classifies 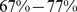 correctly. Specifically, the SnIPRE methods are more sensitive for small (close to zero) and more accurate for extreme valued selection coefficients. The selection coefficients not identified by SnIPRE as significantly different from zero, are generally within 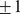 of zero.

**Table 8:** Selection classification for simulations by method.

	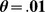 (Drosophila)			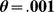 (Human)		
	Dist 1	Dist 2	Dist 3	Dist 1	Dist 2	Dist 3
SnIPRE	0.95	0.92	0.95	0.86	0.72	0.88
B SnIPRE	0.93	0.90	0.93	0.85	0.76	0.86
MKprf ( 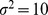 )	0.90	0.83	0.89	0.50	0.45	0.60
MKprf (estimated 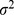 )	0.90	0.83	0.89	0.52	0.42	0.57
MK	0.77	0.67	0.76	0.20	0.12	0.15

Proportion of genes correctly classified under selection where the selection coefficients are from distribution 1, 2 and 3; mutation rate 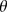.

It is important to note that the increased power of SnIPRE does not rely on the type of selection, since positive, negative, or balancing may affect the MK table counts similarly. We focused here on data simulated with negative and positive selection with constant population sizes, however, SnIPRE will have more power to detect deviations from the neutral expection of 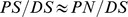 than the MK regardless of the reason. For example, if balancing selection disrupts the 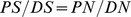 equality to the same extent as some other selection pressure (for example, an average selection coefficient of 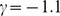 under constant population size, which is simulated here), the relative improvement in SnIPRE over MK would be the same.

**Figure 6:**
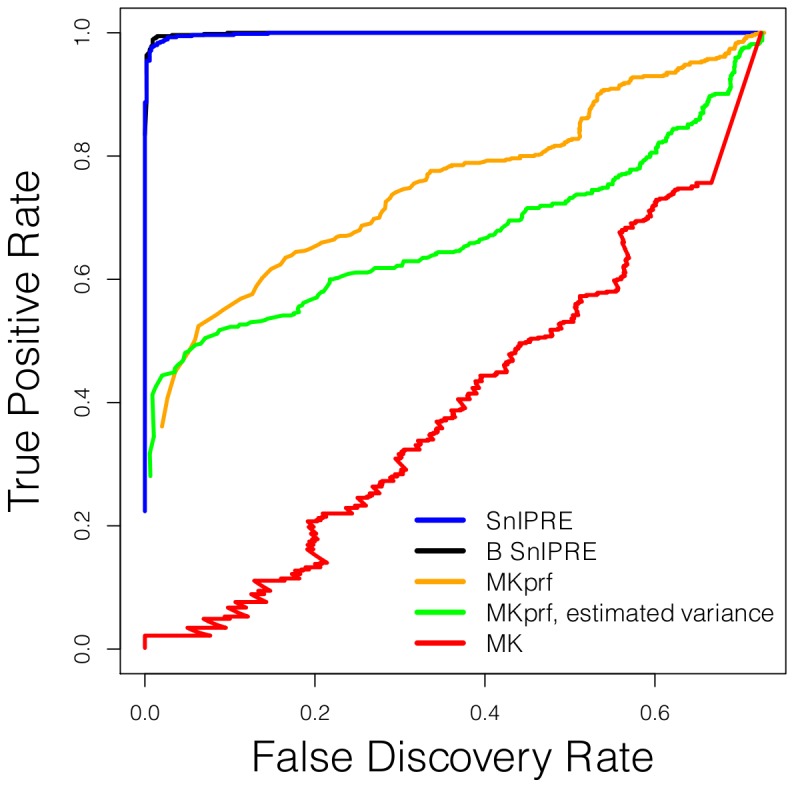
True positive rate versus false discover rate. Results for data set of 2,000 genes, 550 of the genes are under selection with 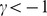 or 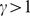.

The methods were also tested on a data set which contained both genes with and genes without mutations under selection (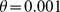, selection strength of at least 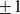, simulation done in PRFREQ). In Figure 6 the true positive rate is plotted versus the false discovery rate. Here we see that at the cutoff needed for the MK statistic to have identified half the genes under selection (TPR = 0.5), approximately half of the discoveries are false (FDR
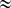
0.5). The MKprf methods offer a dramatic improvement of the MK statistic with a FDR approximately equal to 0.1 at a TPR = 0.5, but the SnIPRE methods offer further improvement with a FDR of zero at TPR = 0.5.

#### Estimation of selection coefficient, γ

**Figure 7:**
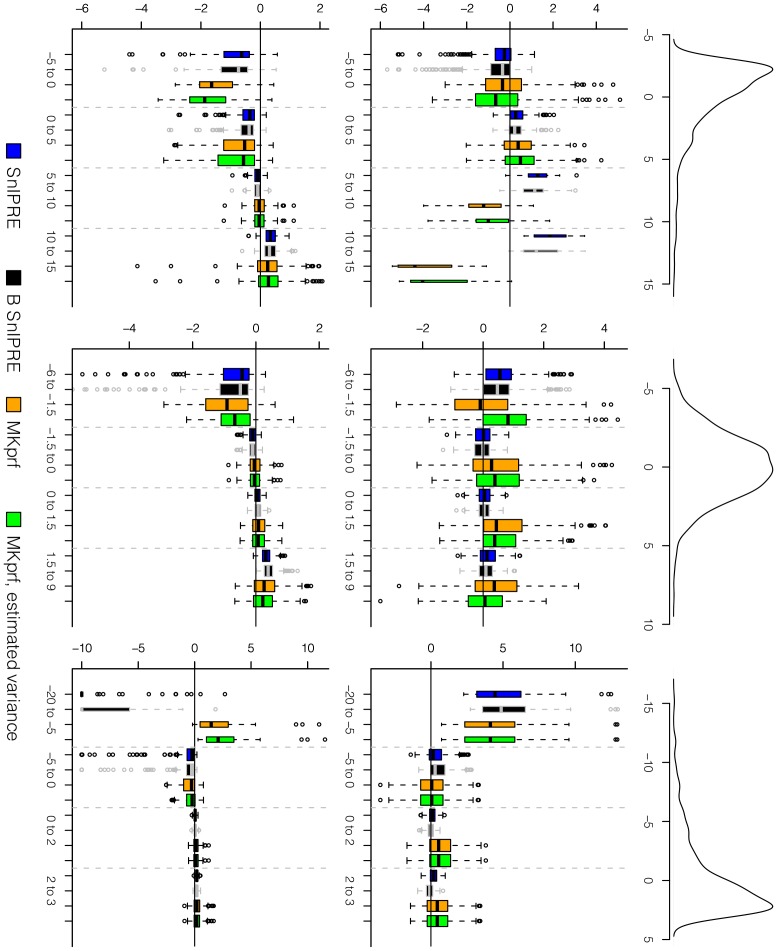
Distribution of residuals for selection coefficient estimates by method. The top row displays the distribution of constraint, the middle row displays residuals for simulations using 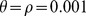; the bottom row displays residuals for simulations using 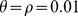. Residuals grouped by true selection strength.

As previously mentioned, the SnIPRE method can be used not only to reject the hypothesis of neutral evolution for a particular gene, but can also be used with additional assumptions to provide estimates of the selection coefficient, 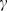. We compare the SnIPRE and MKprf classification success of 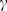 for the PRFREQ simulation data in Figures 4 and 5. The distribution of the differences between the estimates and the true selection coefficent 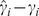 for each method is shown in Figure 7. The SnIPRE methods generally yield reasonable results for genes with selection coefficients from 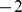 or higher. However, for genes under strong negative selection cell counts are often quite small or zero, and since the cell counts are bounded below by zero it is hard to estimate precisely the extent of negative selection. Because of this, both the SnIPRE methods and MKprf method suffer in precise estimation of negative selection coefficients. However, as seen in Figure 5 the SnIPRE methods still classify these coefficients as negative, whereas MKprf does so for only a fraction of the more extreme cases.

### Application

**Figure 8:**
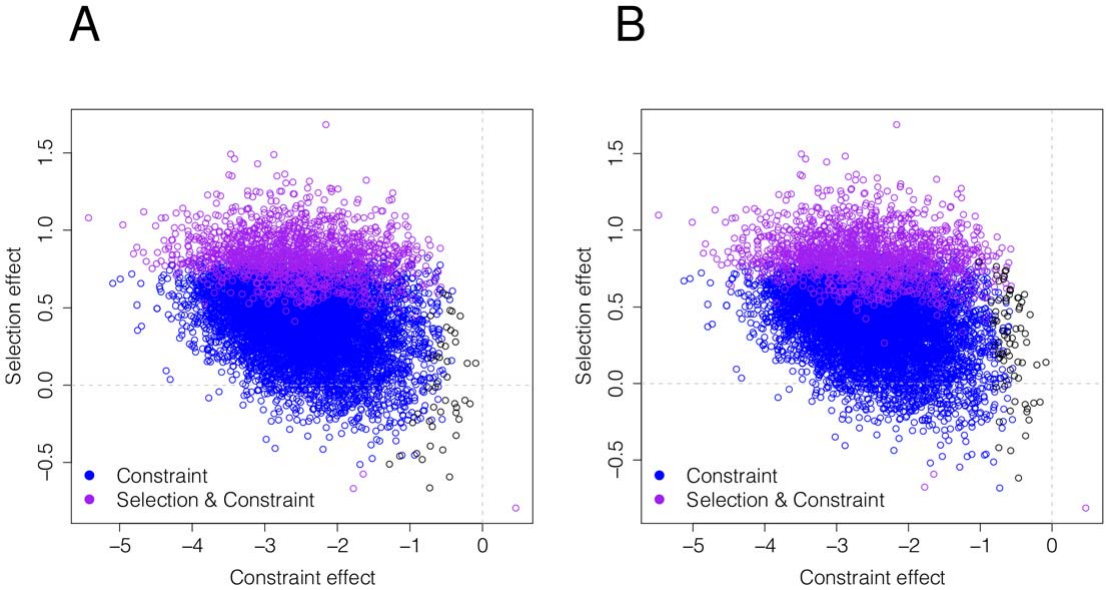
*D. simulans* estimated selection effects and non-synonymous effects for 8,887 genes. Plots A and B shows the estimated selection effects using SnIPRE and B SnIPRE respectively.

We also applied these methods to *Drosophila simulans* data with a *Drosophila melanogaster* outgroup. This data was originally presented by Begun et al [31]. Our results are consistent with others' findings of abundant positive selection among *Drosophila* [32]–[33] [16]. B SnIPRE identifies an additional 613 genes (nearly a 60% increase) with significant evidence of positive selection that were not significant by the traditional MK test using an un-adjusted p-value cutoff of 0.05. We also find evidence of a significant amount of mutational constraint, see Figure 8. These results are consistent with the large effective population size of Drosophila and the strong efficacy of selection. It is important to note when interpreting these results that all the tests discussed here have an underlying assumption that the synonymous sites are under no selection. These synonymous sites act as a baseline, thus conclusions of positive or negative are actually measured relative to the level of selection acting on synonymous sites. For example, if there is selection against unfavored codons, this may artificially inflate the non-synonymous to synonymous ratio and be misinterpreted as positive selection at non-synonymous sites. If codon bias is believed to be widespread amongst the genome, a better indicator of selection levels may be to compare the gene specific effects to the genome average, rather than comparing the sum of these effects to zero.

**Figure 9:**
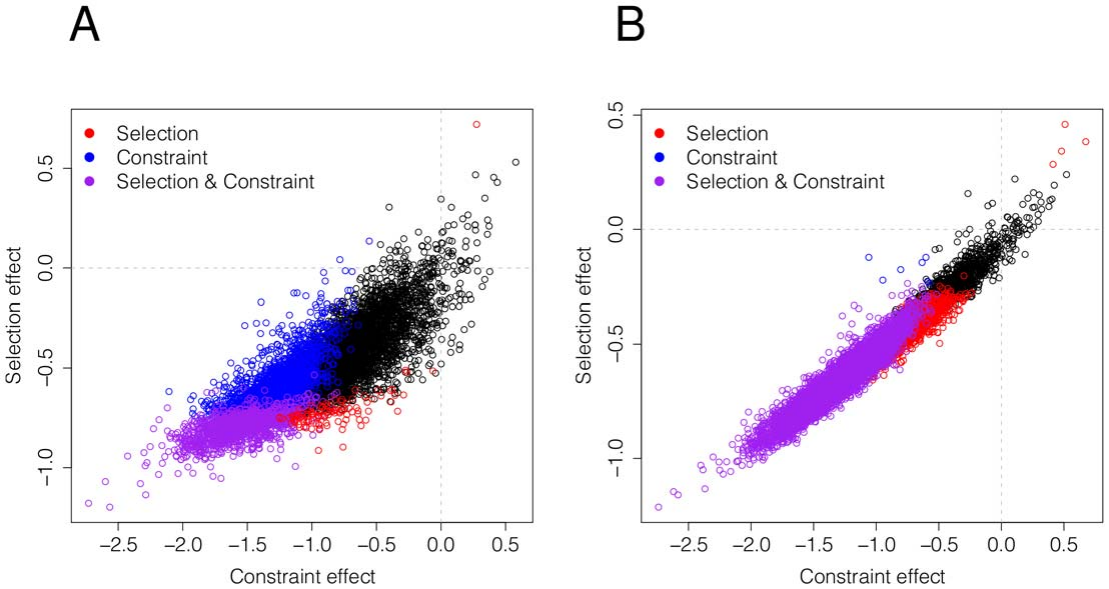
Human estimated selection effects and non-synonymous effects for 11,624 genes. Plots A and B shows the estimated selection effects using SnIPRE and B SnIPRE respectively. B SnIPRE classifies far more genes as having a negative average selection effect, and this difference can be explained in part by the construction of 95% confidence interval versus the credible interval.

In contrast, when we applied SnIPRE to human data, we found few genes with evidence of strong positive selection and an overwhelming signal of negative selection, see Figure 9. This is consistent with our previous interpretation of the results in [10] and [12], where we argued weak negative selection is the predominant mode of selection operating across the majority of human evolutionary history. Again, this is consistent with the small long term 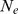 of our species. An implication of this result is that many genes likely harbor mutations of small negative effect that can reach appreciable frequencies.

The application to humans in particular illustrates nicely the improved power in the SnIPRE model to detect genes under strong negative selection (constraint) and recurrent negative selection on mildly deleterious mutations. Because of the relatively low mutation rate in humans, genes under varying degrees of negative selection usually have such low mutation counts in the MK table that the MK test is unable to achieve significance. For example, consider the spermatogenic *Odf2* gene, which plays an important role in sperm morphology and infertility. The MK table counts are as follows: PS = 1, DS = 9, PN = 1, and DN = 1. The MK test is testing the equality 1/9 = 1/1, but failed to reach significance (p-value = 0.32). SnIPRE, however, found significant evidence of negative selection, as well as mutational constraint. The SnIPRE estimated selection effect for this particular gene was 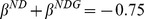 (significantly different from zero, and lower than the genomic average of 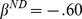), and the estimated reduction in non-synonymous mutations was also quite strong, 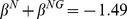 (compared to a genomic average of 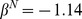). From here we can conclude that there is significant evidence of selection, and additionally, there may be evidence of mutational constraint, or purifying selection, as we are observing significantly fewer non-synonymous mutations than expected. It is difficult to interpret the significance of the constraint, however, without first estimating the strength of negative selection. This is because the strength of selection also influences the expected number of non-synonymous mutations. If we are willing to accept the additional assumptions of the PRF framework, then using the relationship defined in (7) and (8) we estimate the average selection coefficient acting on this gene to be equal to 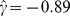, and the estimated proportion of mutations that are non-lethal in this gene to be 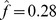 (a proportion which is found to be significantly different from 1). Under the PRF framework the SnIPRE model also tells us that the gene effect for *Odf2*, 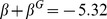 may be interpreted as mutation rate of 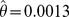 mutations per generation, per site (slightly higher than the estimated genomic average estimated from this data of 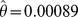); and the estimated divergence effect, 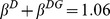, leads to an estimated scaled coalescence time for this gene at 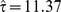 (slightly higher than the genomic average estimated here of 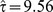).

The *BRC2* gene, associated with breast cancer and important for DNA repair, is another illustration of a case where examining the individual MK table we are unable to find significant evidence of selection. However the SnIPRE model indicates a significant amount of mutational constraint, indicating strong negative selection. The MK table for this gene has PS = 13, DS = 16, PN = 9, and DN = 17. While there is little evidence of negative selection (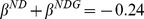, not significantly different than zero), the SnIPRE model indicates evidence for mutational constraint (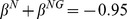). From the MK table alone we would not see this as the total synonymous and non-synonymous mutations are similar. However, considered with the additional information that the number of non-synonymous sites sampled was nearly three times the number of synonymous sites sampled, the SnIPRE model in the PRF framework estimates the proportion of mutations that are non-lethal to be 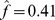, significantly different than one. The average mutation rate for this gene is estimated to be 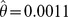 and a more recent coalescent time of 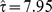.

Due to the overwhelming evidence of negative selection and constraint in humans, signatures of positive selection are difficult to detect even with the increase in power with the SnIPRE framework. B SnIPRE detects only 4 genes under positive selection not identified by the traditional MK statistic, which identifies 10 genes. For this reason it may be informative to consider the effect of selection on a gene *relative* to the genome-wide average. Because the selection effect represents the average effect of selection on that gene throughout time, it may represent an average of both positive and negative selection forces. Assuming a model where we can interpret the the sign of the selection effect as indictive of the direction of selection, genes with selection effects significantly higher than the genome-wide average will have had either more positive selection or less negative selection acting on them than the typical gene. For example, in this data set B SnIPRE identifies 628 genes with selection effects significantly higher than the genome-wide average of −0.60.

### Conclusions

The SnIPRE framework models MK table data in a way consistent with population genetic theory and with minimal assumptions on the demographic model may reject the neutral theory. However, just as with the traditional MK test, conclusions about type of selection (positive, negative, or balancing) require further assumptions. The parameters of the SnIPRE model are easily interpreted and can be effectively used to estimate the affects of selection, constraint, divergence time, and mutation rate on genome-wide patterns of variation on a gene-by-gene basis. Effects may be readily evaluated in the absolute, or relative to the genome-wide estimates.

The simulations provided here illustrate the significant increase in power over the traditional MK test that the SnIPRE model provides, while maintaining a low false positive rate. This makes sense since we are using genome-wide data to improve our estimate of the influence of mutation rate, species divergence time, constraint, and selection effects. The fixed effects reflect genome-wide averages of these effects; the random effects reflect the gene-by-gene variation in the influence of these forces and provide estimates of this variation with James-Stein-type shrinkage. Both the empirical Bayes and fully Bayesian implementation borrow strength across genes to improve estimates of the parameters of interest. The success of the method in simualtions, as well as the consistency of the *Drosophila* and human-chimp results with other findings corroborates the legitimacy of this methodology in this setting.

When the assumptions of the PRF are met, our simulations indicate the method provides estimates of the selection coefficient as un-biased as the more parametric method MKprf, and with generally smaller confidence intervals. While in this paper we have focused on the interpretation of SnIPRE parameters in the PRF framework, we believe an extension of the model could be used in another framework which allows for arbitrary dominance. One such framework is described in Williamson et al [34] in which the dominance parameter is estimated based on additional information from the site frequency spectrum. However, as with any method that makes conclusions about strength and directionality, such as MKprf or 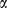, in order to asses the type of selection assumptions would need to be made about effective population size changes and their timing.

In the future, we will explore the impact of varying recombination rate on the accuracy of parameter estimates and, in turn, the efficacy of natural selection in weeding out deleterious alleles while promoting favorable mutations to high frequency.
